# Test your knowledge and understanding

**Published:** 2017-02-10

This page is designed to help you to test your own understanding of the concepts covered in this issue, and to reflect on what you have learnt. We hope that you will also discuss the questions with your colleagues and other members of the eye care team, perhaps in a journal club. To complete the activities online – and get instant feedback – please visit **www.cehjournal.org**

**Table:** 

**1.**	**Ocular surface disease may affect the following:**	**Tick all that apply**
**a**	Conjunctiva	□
**b**	Tear film	□
**c**	Iris	□
**d**	Cornea	□
**e**	Eyelid margins	□
**2.**	**What is important in the treatment of blepharoconjunctivitis?**	**Tick all that apply**
**a**	Systemic prednisolone	□
**b**	Tarsorrhaphy	□
**c**	Warm compresses to the eyelids	□
**d**	Topical atropine	□
**e**	Mechanical debridement of eyelash crusts	□
**3.**	**Dry eye syndrome:**	**Tick all that apply**
**a**	Is more common with increasing age	□
**b**	Is improved by a hot, dry atmosphere	□
**c**	Can cause punctate epithelial erosions	□
**d**	Can be treated with artificial tears	□
**e**	May result in Mooren's ulcer	□
**4.**	**Which of these statements are true?**	**Tick all that apply**
**a**	Stevens Johnson Syndrome may be associated with HIV positive status	□
**b**	Epiphora means a dry eye	□
**c**	Vernal keratoconjunctivitis is associated with keratoconus	□
**d**	Herpes zoster ophthalmicus may cause corneal anaesthesia	□
**e**	Alkali burns to the eye are usually more serious than acid burns	□
**5.**	**The following are useful diagnostic tests in ocular surface disease:**	**Tick all that apply**
**a**	Direct ophthalmoscopy	□
**b**	Slit lamp examination of the tear film	□
**c**	Fluorescein staining of the cornea	□
**d**	Testing for corneal sensation	□
**e**	Schirmer's test	□

## ANSWERS

1. Answers **a, b, d** and **e.** As the name indicates, the surface of the eye can be affected, but not the deep tissues such as uvea (iris) and retina.
2. Answers **c** and **e.** Hot bathing and removal of any debris at the base of the eyelashes are important, together with eyelid massage.
3. Answers **a, c** and **d** are correct. Hot dry atmospheres make dry eye symptoms worse. Dry eye syndrome does not cause Mooren's ulcer.
4. **All the answers are true except b**. Epiphora means a watering eye. Note: Stevens-Johnson syndrome may be due to an adverse reaction to some medications.
5. **All are true except a**.

## REFLECTIVE LEARNING

Visit **www.cehjournal.org** to complete the online ‘Time to reflect’ section.

